# Innervated mouse pancreas organoids as an *ex vivo* model to study pancreatic neuropathy in pancreatic cancer

**DOI:** 10.1016/j.xpro.2021.100935

**Published:** 2021-11-17

**Authors:** H. Erdinc Besikcioglu, Ümmügülsüm Yurteri, Enkhtsetseg Munkhbaatar, Linhan Ye, Fangfang Zhang, Alessandra Moretti, Carmen Mota Reyes, Candan Özoğul, Helmut Friess, Güralp O. Ceyhan, Rouzanna Istvanffy, Ihsan Ekin Demir

**Affiliations:** 1 Department of Surgery, Klinikum rechts der Isar, Technical University of Munich, School of Medicine, Munich, Germany; 2 German Cancer Consortium (DKTK), Partner Site Munich, Munich, Germany; 3 CRC 1321 Modelling and Targeting Pancreatic Cancer, Munich, Germany; 4 Department of Histology and Embryology, Institution of Health Sciences, Gazi University, Ankara, Turkey; 5 Department of Internal Medicine I, Klinikum rechts der Isar, Technical University of Munich, School of Medicine, Munich, Germany; 6 Department of Histology and Embryology, Faculty of Medicine, University of Kyrenia, Kyrenia, Cyprus; 7 Department of General Surgery, HPB-Unit, School of Medicine, Acibadem Mehmet Ali Aydinlar University, Istanbul, Turkey; 8 Else Kröner Clinician Scientist Professor for Translational Pancreatic Surgery

**Keywords:** Cell culture, Cell isolation, Cancer, Model Organisms, Neuroscience, Stem Cells, Cell Differentiation, Organoids

## Abstract

Pancreatic cancer is characterized by bi-directional interactions between pancreatic cancer cells and stromal cells including neural cells. The absence of neural cells in pancreatic organoids limits the investigation of cell- cell interaction and tumor innervation. This protocol describes how to generate innervated wild type (WT) and Kras^+/LSLG12D^ Trp53^fl/f l^p48^+/Cre^ (KPC) murine pancreatic organoids. To specifically investigate neurogenesis, organoids are co-cultured with iPSCs-derived neural crest cells, while co-culture with dorsal root ganglia explants is used for comparing organoids with mature neurons.

For complete details on the use and execution of this protocol, please refer to Huch et al. (2013), Boj et al. (2015), and Demir et al. (2014).

## Highlights

• Protocol for the generation of “innervated” pancreas organoids
• Generation of innervated wildtype (WT) and cancer (KPC) murine pancreas organoids
• Innervation enables investigation of neuron – cancer cell interactions in PDAC
• NCCs-pancreas organoid co-cultures are also useful for studying neurogenesis in pancreas development

## Graphical abstract

**Figure:**
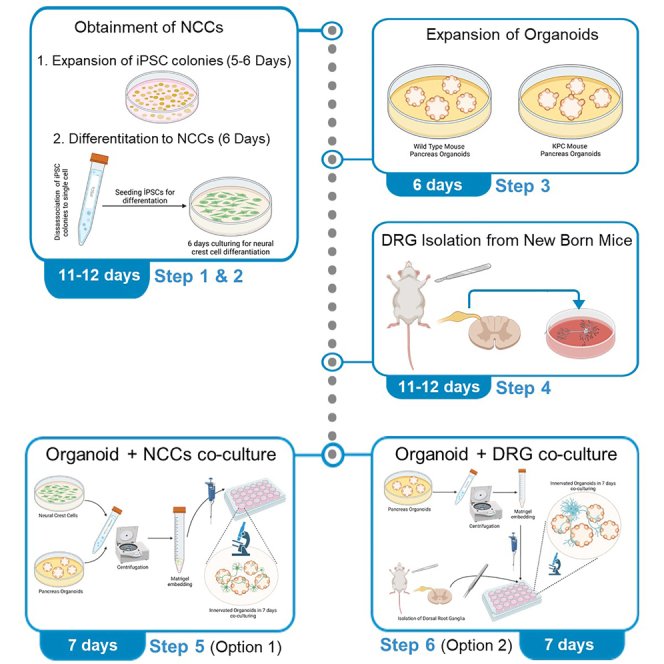

## Before you begin

All solutions must be prepared before starting the experiments, according to recipes in the materials and equipment section. It is also possible to prepare some of the solutions and store as indicated in the footnotes of recipes.

### Preparations for iPSC culture and NCC differentiation

**Timing: 2 h**

***Note:*** Please read the manufacturer’s instructions for the preparation and storage of Tesr_E8 Medium® and Stemdiff Neural Crest Differentiation Kit®. Both media are delivered as two separated bottles as basic medium and supplement. Basic medium and supplement must be mixed and aliquoted. Both media can be stored at +4°C up to one week and must be stored at −20°C for longer time frames.

1. Take out Tesr_E8 medium aliquot for iPSC culture and/or Neural Crest Differentiation medium aliquot for NCC differentiation from the +4°C around 2 h before starting and let them warm up to 20°C–25°C.

**CRITICAL:** Do not warm Tesr_E8 medium and Neural Crest Differentiation medium in 37°C water bath.

2. Put Matrigel hESC-Qualified Matrix aliquots on ice and wait it to liquefy for ~1 h. Aliquot size can be between 150–200 μL for 12 mL coating medium. Refer to certificate of analysis provided by manufacturer as dilution factor for recommended aliquot amount.

3. Coating the plates.
  a. Prepare 12 mL of coating medium.
    i. Fill a 15 mL conical tube with 12 mL ice-cold DMEM/F12 medium.
    ii. Add appropriate amount of Matrigel hESC-Qualified Matrix, the needed amount of matrix for 25 mL coating medium is indicated as dilution factor in certificate of analysis provided by the manufacturer (e.g., 309 μL for 25 mL).
      **CRITICAL:** The dilution factor varies from lot to lot. Always check certificate of analysis on manufacturer’s webpage when opening a new vial.
    iii. Mix the coating medium by pipetting up and down a few times with a 10 mL serological pipette attached on pipette boy.
  b. Add 6 mL of coating medium to 100 mm petri dish or 1 mL coating medium/per well to 6-well plate. Let it sit for a minimum of 1 h at 20°C–25°C under a class II biosafety cabinet.
    ***Optional:*** It is possible to prepare more plates. Tightly seal them with parafilm to avoid evaporation and store them at 4°C up to one week.

4. Warm up the sterile PBS in 37°C water bath.

**CRITICAL:** iPSCs tends to differentiate when they are out of the incubator more than 10 min. Warming up all the solutions before taking the cells out from the incubator is important for the time management.

### Preparation for organoid culture

**Timing: 1 day for warming plates, 20 min for preparing media and reagents**

5. Put 24-well adhesion plates in a 37°C incubator at least 24 h before starting.
6. Take out 500 μLMatrigel Basement Membrane Matrix (BMM) aliquot from −20°C and keep on ice for one and half hours until it is completely liquefied.
7. Take out the mEGF, mNoggin, hFGF-10, hGastrin-I, B27 Supplement, N-acetylcysteine, Nicotinamide and R-Spondin1 conditioned medium aliquots from −20°C and thaw on the ice and thaw A83-01 at 20°C–25°C.
8. Prepare 500 mL mouse organoid basic medium.
  a. Open a new bottle of Advanced DMEM/F-12 and remove 15 mL.
  b. Add 5 mL GlutaMAX (100**×**) solution by using a5 mL serological pipette attached on a pipette boy.
  c. Add 5 mL HEPES (1 M) solution by using a5 mL serological pipette attached on a pipette boy.
  d. Add 5 mL Penicillin-Streptomycin (10,000 U/mL) solution by using a5 mL serological pipette attached on a pipette boy.
9. Prepare 20 mL mouse organoid feeding medium.
  a. Add 18 mL mouse organoid basic medium in a 50 mL conical tube.
  b. Add 20 μL mEGF (1000**×**).
  c. Add 20 μL mNoggin (1000**×**).
  d. Add 20 μL hFGF-10 (1000**×**).
  e. Add 20 μL hGastrin-I (1000**×**).
  f. Add 20 μL A83-01 (1000**×**).
  g. Add 50 μL N-acetylcysteine (400**×**).
  h. Add 200 μL Nicotinamide (100**×**).
  i. Add 400 μL B27 Supplement (50**×**).
  j. Add 2 mL R-Spondin1 conditioned medium.
10. Warm up the mouse organoid feeding medium in a 37°C water bath.

### Preparation for DRG isolation

**Timing: 1 day for warming well plates, 10 min for preparing media and reagents**

11. Put 24-well plates in a 37°C incubator at least 24 h before starting.
12. Prepare DRG collection medium.
  a. Open a new bottle of 500 mL MEM and take out 400 μL.
  b. Add 400 μL Gentamicin (50 mg/mL).
13. Clean a dissection hood and stereo microscope with 70% Ethanol.
14. Prepare DRG collection plate.
  a. Fill an ice bucket with ice and flatten the surface.
  b. Place a 35 mm petri dish into a 100 mm petri dish and put on the ice.
  c. Fill the 35 mm petri with 2–3 mL DRG collection medium.

### Preparation for Co-culture

**Timing: 1 day for warming well plates, 2 h for thawing Matrigel, 20 min for preparing media and reagents**

15. Put 24-well plates into 37°C incubator at least 24 h before starting.
16. Take out the Matrigel BMM aliquots from −20°C and keep on ice to liquefy.
17. Prepare 20 mL co-culture medium.
  a. Fill a conical tube with 20 mL mouse organoid basic medium.
  b. Add 50 μL N-Acetylcysteine (400**×**).
  c. Add 400 μL B27 Supplement (50**×**).

***Note:*** Add 20 μL Y-27632 (1000**×**) for the first day of establishing co-cultures.

## Key resources table

**Table:** 

REAGENT or RESOURCE	SOURCE	IDENTIFIER
**Chemicals, peptides, and recombinant proteins**		

TeSR-E8	Stemcell Technologies	Cat#05990
STEMdiff Neural Crest Differentiation Kit	Stemcell Technologies	Cat#08610
DMEM/F-12 with 15 mM HEPES and sodium bicarbonate, without L-glutamine	Sigma-Aldrich	Cat#D6421
Gentle Cell Dissociation Reagent (GCDR)	Stemcell Technologies	Cat#07174
Matrigel Growth Factor Reduced (GFR) Basement Membrane Matrix, Phenol Red-free, LDEV-free	Corning	Cat#356231
Matrigel hESC-Qualified Matrix, LDEV-free	Corning	Cat#354277
DPBS without CaCl_2_, without MgCl_2_	Sigma-Aldrich	Cat#D8537
Y27632	Miltenyi Biotec	Cat#130-103-922
Advanced DMEM/F-12	Thermo Fisher/Gibco	Cat#12634010
GlutaMAX 100**×**	Thermo Fisher/Gibco	Cat# 35050061
HEPES (1 M)	Thermo Fisher/Gibco	Cat#15630080
Penicillin-Streptomycin (10,000 U/mL)	Thermo Fisher/Gibco	Cat#15140122
N-Acetylcysteine	Sigma-Aldrich	Cat#A9165-5G
Nicotinamide	Sigma-Aldrich	Cat#N0636-100G
Recombinant Murine Noggin	PeproTech	Cat#250-38
EGF Recombinant Mouse Protein	Thermo Fisher/Gibco	Cat#PMG8043
Recombinant Human FGF-10	PeproTech	Cat#100-26
Gastrin I (human)	Tocris	Cat#3006
A83-01	Tocris	Cat#2939
B27 supplement 50**×** (serum-free)	Thermo Fisher/Gibco	Cat#17504044
Minimum Essential Medium (MEM)	Thermo Fisher/Gibco	Cat#11095080
Buffer RLT	Qiagen	Cat# 79216
2-Mercaptoethanol	Sigma	Cat#63689
Trypan Blue Solution, 0.4%	Thermo Fisher/Gibco	Cat#15250061
Bovine Serum Albumin solution 30% in DPBS, sterile	Sigma-Aldrich	Cat#A9576-50ML
Gentamicin solution 50 mg/mL	Sigma-Aldrich	Cat#G1397

**Experimental models: Cell lines**		

Mouse: C57BL/6	Charles River	n/a
Cell line: BIONi014-A hiPSC	EBiSC	Cat#66540265
HA-R-Spondin1-Fc 293 T	Trevigen	Cat#3710-001-01

**Other**		

Class II Biological Safety Cabinet	Thermo Scientific	Cat#51023654
CO2 Incubator	n/a	n/a
Refrigerated Centrifuge	n/a	n/a
37°C Water Bath	n/a	n/a
Pipette Boy		n/a
Micro pipette set	Eppendorf Research	Cat#3120000909
Inverted microscope	n/a	n/a
Hemocytometer	Sigma	Cat#Z359629
Clean bench cabinet	n/a	n/a
Stereo microscope	n/a	n/a
Ultra fine micro vannas scissors (straight)	n/a	n/a
Ultra fine micro vannas scissors (curved)	n/a	n/a
Ultra fine micro forceps (straight)	n/a	n/a
Ultra fine micro forceps (curved)	n/a	n/a
15 mL Conical tube	Greiner bio-one	188261
50 mL Conical tube	Greiner bio-one	210270
6-well plate	Corning	353046
24-well plates	Corning	353047
35 mm petri dish	Corning	430165
100 mm dish	Corning	353003
Cell scraper	Sarstedt	83.1832
1.5 mL tubes	Eppendorf	0030 123.328
10 μL unfiltered sterile pipette tip	Starlab	S1110-3700
10 μL filtered sterile pipette tip	Starlab	S1120-3810-C
100 μL filtered sterile pipette tip	Starlab	S1120-1840-C
200 μL filtered sterile pipette tip	Starlab	S1120-8810-C
1000 μL filtered sterile pipette tip	Starlab	S1126-7810
5 mL Serological pipette	Greiner Bio-one	606180
10 mL Serological pipette	Greiner Bio-one	607160
25 mL Serological pipette	Greiner Bio-one	760180
50 mL Serological pipette	Greiner Bio-one	768180

## Materials and equipment

**Table:** Mouse organoid basic medium

Reagent	Final concentration	Amount
Advanced DMEM/F-12	n/a	485 mL
Glutamax (100**×**)	1**×**	5 mL
Penicillin-Streptomycin (10,000 U/mL)	1000 U/mL	5 mL
HEPES (1 M)	10 mM	5 mL
**Total**	**n/a**	**500 mL**

It can be stored at 4°C up to 1 month.

**Table:** Mouse organoid feeding medium

Reagent	Final concentration	Amount
Mouse organoid basic medium	n/a	18 mL
1000**×** mEGF (50 μg/mL)	1**×** (0.05 μg/mL)	20 μL
1000**×** hFGF-10 (100 μg/mL)	1**×** (0.1 μg/mL)	20 μL
1000**×** A83-01 (0.21 mg/mL)	1**×** (0.21 μg/mL)	20 μL
1000**×** mNoggin (100 μg/mL)	1**×** (0.1 μg/mL)	20 μL
1000**×** hGastrin-I (0.021 mg/mL)	1**×** (0.021 μg/mL)	20 μL
400**×** N-acetylcysteine (81.5 mg/mL)	1**×** (0.2 mg/mL)	50 μL
100**×** Nicotinamide (122 mg/mL)	1**×** (1.22 mg/mL)	200 μL
50**×** B27 Supplement	1**×**	400 μL
10**×** RSpondin-1 conditioned medium^a^	1**×**	2 mL
**Total**	**n/a**	**20 mL**

It can be stored at 4°C up to 14 days

***Note:***^a^ R-Spondin-1 conditioned medium was produced from HA-R-Spondin1-Fc 293 T Cell Line which is commercially available from Trevigen (Cat# 3710-001-01).

**Table:** DRG collection medium

Reagent	Final concentration	Amount
MEM	n/a	500 mL
Gentamicin solution (50 mg/mL)	40 μg/mL	400 μL
**Total**	**n/a**	**500 mL**

It can be stored at 4°C up to 1 month.

**Table:** Co-culture medium

Reagent	Final concentration	Amount
Mouse organoid basic medium	n/a	19.53 mL
50**×** B27 Supplement	1**×**	400 μL
400**×** N-acetylcysteine (81.5 mg/mL)	1**×** (0.2 mg/mL)	50 μL
Y27632 (10 mM)^a^	10 μM	20 μL
**Total**	**n/a**	**20 mL**

It can be stored at 4°C be used up to 10 days.

***Note:***^a^ Y27632 is needed for the first establishment of the co-cultures. There is no need to add Y27632 following media changes.

## Step-by-step method details

### Culturing human induced pluripotent stem cells (hiPSCs) for differentiation

**Timing: 45 min for passaging, for culturing 5–6 days**

This step describes how to grow hiPSCs on Matrigel coated 100 mm dishes. One confluent 100 mm dish has approximately 6–8∗10^6^ cells. Prepare minimum three 100 mm dishes for NCC differentiation step (2∗10^6^ cell/mL/well hiPSCs are needed for NCCs differentiation in a 6- well plates.).

1. Passaging hiPSCs colonies
  a. Check for differentiated areas under an inverted microscope and mark the differentiated regions (Figures 6C and 6D.) with a permanent marker
  b. Take the dish into the sterile hood and scratch and detach the differentiated cell areas using a 1000 μL pipette tip
  c. Remove old media and differentiated cells from the culture dish using an aspirating system.
  d. Gently and slowly add 12 mL pre-warmed sterile PBS on the side of the dish.
  e. Remove PBS using aspirator and add 6 mL of pre-warmed gentle cell disassociation reagent (GCDR) and incubate for 7 min at 20°C–25°C.
  f. During the incubation in step e, prepare at least three culture dishes which were previously coated for seeding hiPSCs (prepared at Step 3/b in before you beginsection), by aspirating the coating medium and adding 11.5 mL pre-warmed TesR_E8 medium.
  g. Remove the GCDR and add 4 mL pre-warmed TesR_E8 medium.
  h. Gently scrape and detach the colonies with a cell scraper.
  i. Pipette up and down 2 times with a 5 mL serological pipette to break the colonies into smaller pieces
  j. Add 0.5 mL TesR_E8 hiPSCs colonies suspension into the culture dishes prepared in Step 1/f
  k. Put dishes into an incubator (37°C and 5% CO_2_), and gently shake right to left and backward to forward for a few times

**CRITICAL:** Do not open the incubator and the cells for 24 h.

2. Daily media change with TesR_E8
  a. Slowly remove the old medium.
    **CRITICAL:** Do not remove all of the old media. Leave 1–2 mL of the old media to avoid drying out the cells.
  b. Add 12 mL pre-warmed fresh TesR_E8
    ***Note:*** Media must be changed every day for 5–6 days until iPSC colonies reach 70–80% confluency has compact center (Figure 6).

### Neural crest cell differentiation from hiPSCs

**Timing: 30 min for plating the hiPSCs, 6–7 days for differentiation**

This step describes how to disassociate hiPSCs colonies to single cells and the differentiation to neural crest cells in Matrigel coated 6-well plates.

3. Preparing single hiPSCs suspension.
  a. Repeat from Step1/a and Step1/b to remove differentiated cells.
    **CRITICAL:** It is very important to use undifferentiated compact hiPSCs colonies with smooth border. See Figure 6 in expected outcomes section for optimal iPSCs colonies.
  b. Aspirate old media and differentiated cells.
  c. Gently and slowly add 12 mL pre-warmed PBS on the side of the dish.
  d. Aspirate the PBS and gently add 6 mL pre-warmed GCDR on the side of the dish.
  e. Incubate at 20°C–25°C for 10 min.
  f. Detach the colonies by scraping with a cell scraper.
  g. Transfer the cell suspension into a 15 mL conical tube.
  h. Wash the dish with 6 mL warm DMEM/F-12 with 15 mM HEPES and complete the cell suspension up to 12 mL into the 15 mL conical tube.
  i. Disassociate colonies to single cells by pipetting the cell suspension up and down 8–10 times with a 5 mL serological pipette.
    Take 200–300 μL in a 1.5 mL tube for cell counting and count cells using a hemocytometer or an automated cell counter.
  j. Centrifuge the cell suspension at 300 *g* for 5 min at 20°C–25°C.
  k. Remove the supernatant and resuspend the pellet with appropriate amount of STEMdiff™ Neural Crest Differentiation Medium + 10 μM Y27632 to final concentration of 1∗10^6^ cell/mL.
    ***Optional:*** The NCCs can be also seeded on coverslips to check negativity of early neuro ectodermal marker PAX6 and positivity of neural crest marker SOX10 by staining.
    **CRITICAL:** Addition of 10 μM Y27632 is very important for cell survival and to inhibit spontaneous differentiation on the first day of single cell passaging of iPSCs
  l. Aspirate coating media from pre-coated 6-well plates and add 2 mL cell suspension per well (corresponding to 2∗10^6^ cells) in a 6-well plate.
  m. Put dishes into the incubator (37°C and 5% CO_2_) and gently shake right to left and backward to forward for a few times
    **CRITICAL:** Do not touch the incubator and the cells for 24 h.

4. Daily media change with STEMdiff™ Neural Crest Differentiation Medium
  **CRITICAL:** Media must be chadnge every day during 6 days of differentiation (Figure 7).
  a. Slowly remove the old media.
    **CRITICAL:** Do not remove all the old media to avoid drying out the cells.
  b. Add 2 mL pre-warmed fresh STEMdiff™ Neural Crest Differentiation Medium. Addition of Y-27632 is not needed after the first day of the culture.

### Culturing organoids

**Timing: 1 h for plating, 6 d for culturing**

This step describes how to passage mouse pancreatic organoids (1:6 ratio) for culturing them until the co-culture day.

**CRITICAL:** Timing is important for obtaining ~4∗10^4^ cell/mL (individual cell number in one 80% confluent well of 24 well plate) organoids on the day of the co-culture. It takes about 6 days to reach 80% confluency in organoid cultures (Figure 8).

5. Passaging Organoids.
  a. Aspirate the old medium from 1 well of 24-well plate.
  b. Add 1 mL ice-cold mouse organoid basic medium.
  c. Detach the Matrigel BMM dome by scratching the bottom of well with pipette tip and transfer organoids into a new 15 mL falcon tube.
    ***Note:*** Coating pipette tip with BSA by pipetting in 30% sterile BSA solution a few times, prevents the organoid loss due to adhesion on surface of pipette tip.
  d. Fill the tube up to 12 mL with ice-cold mouse organoid basic medium.
  e. Centrifuge at 200 *g* and 4°C for 5 min.
  f. Remove ~10 mL supernatant to leave ~1,5 - 2 mL with the pellet.
  g. Pipette up and down 9 times with P-1000 pipette with a 10 μL unfiltered tip attached to a 1000 μL filtered tip.
  h. Fill the tube up to 12 mL with ice-cold mouse organoid basic medium.
  i. Centrifuge at 200 *g* and 4°C for 5 min.
  j. Carefully remove the supernatant and resuspend the pellet with 300 μL Matrigel BMM.
    **CRITICAL:** Always keep the Matrigel BMM and samples on ice to avoid solidification of Matrigel above 10°C.
  k. Make 50 μL Matrigel BMM organoid suspension droplets per well in 6 wells of a pre- warmed 24-well plate.
  l. Incubate at 37°C and 5%CO_2_ for 10 min.
  m. Add 500 μL pre-warmed mouse organoid feeding medium per well.
    **CRITICAL:** Mouse organoid feeding medium must be warm to avoid dissolving of the Matrigel BMM.
  n. Change the media with 500 μL pre-warmed mouse organoid feeding medium per well after 3 days.

### Co-culturing neural crest cells and organoids for innervated organoids

**Timing: 30 min for preparing organoids, 15 min for preparing NCCs, 30 min for plating the co-culture**

This step describes how to disrupt the organoids into smaller fragments, to harvest neural crest cells, to seed NCCs and organoids and the co-culture to form innervated pancreas organoids.

**CRITICAL:** Cell number ratio between organoids and NCCs is important for the efficiency of neural differentiation and innervation of organoids. A ratio of 5000 cells/mL organoids (individual number of cells that compose organoids) and 10000 cells/mL NCCs was determined as optimal in our settings (24-well plate with 50 μL/well of Matrigel BMM droplet), however this ratio may be changed depending on the features of the used organoids and iPSC lines.

***Note:*** We advise to count single cell number in one 80% confluent well of organoids from 24 well plate for estimating the cell number of your organoids. (Figure 8)

6. Preparing Organoids for co-culture
  a. Aspirate the old media from 1 well of 24-well plate.
    ***Note:*** One well of pancreatic organoids from a 24-well plate has ~4∗10^4^ cell/mL (Figure 8). Therefore, 1 to 8 splitting ratio is recommended to obtain 5,000 cell/mL organoids in co-culture.
  b. Add 1 mL ice-cold mouse organoid basic medium.
  c. Detach the Matrigel BMM dome by scratching with pipette tip and transfer organoids into a new 15 mL falcon tube.
  d. Fill the tube up to 12 mL with ice-cold mouse organoid basic medium.
  e. Centrifuge at 200 *g* and 4°C for 5 min.
  f. Remove ~10 mL supernatant and leave ~1.5–2 mL with the pellet.
  g. Pipette up and down 7 times with P-1000 pipette with a 10 μL unfiltered tip attached to a 1000 μL filtered tip.
    **CRITICAL:** Avoid over disassociation, which decreases organoid viability.
  h. Keep organoids on ice until NCCs are prepared.

7. Preparing NCCs for co-culture
  a. Remove the old medium.
  b. Slowly add 2 mL per well pre-warmed PBS on the side of the wells.
  c. Aspirate the PBS and add 1 mL GCDR per well.
  d. Incubate the plate at 37°C and 5% CO_2_ for 10 min.
  e. Transfer the cells into a 15 mL conical tube.
  f. Wash the wells with 2 mL mouse organoid basic medium to collect the rest of the cells and transfer into the 15 mL conical tube.
  g. Pipette up and down 5–10 times to completely disassociate clumps to single cells with a 10 mL serological pipette attached on a pipette boy
    i. Take 500 μL cell suspension into a 1,5 mL tube to count the cells during the next step.
  h. Centrifuge the 15 mL conical tube filled with cell suspension at 200 *g* and 4°C for 5 min.
  i. Remove the supernatant and resuspend the cell pellet with mouse organoid basic medium. The estimated cell number in this tube is 6–8∗10^6^ cell/mL. According to your counting in Step 7/g-i, make serial dilution with of mouse organoid basic medium to a final concentration of 1∗10^4^ cell/mL.

8. Preparing NCCs and Organoid Co-culture
  a. Transfer 8 mL of NCCs suspension which has 1∗10^4^ cell/mL (total 8∗10^4^ cell) into the 15 mL conical tube, which contains organoid fragments prepared at Step 6/h to get final volume 10 mL.
    ***Optional:*** The rest of the NCCs should be centrifuged, resuspended in RLT buffer and stored at −80°C for further quantification of neural crest specific genes, such as *Snail-2* and *SOX10*.
    ***Note:*** Add 10 μl β-mercaptoethanol per 1 ml RLT buffer for denaturation of RNases. β-mercaptoethanol is very toxic. Mix it in a fume hodd and wear protective clothes.
  b. Centrifuge at 200 *g* and 4°C for 5 min.
  c. Carefully remove as much supernatant as possible.
  d. Resuspend the pellet with 400 μL Matrigel BMM.
    **CRITICAL:** Always keep Matrigel BMM and samples in ice to avoid solidification of Matrigel above 10°C.
  e. Drop 50 μL of the Matrigel BMM organoid/NCCs suspension droplets per well in 8 wells of pre-warmed 24-well plate.
  f. Incubate at 37°C and 5% CO_2_ for 10 min.
  g. Add 500 μL pre-warmed co-culture media with 10 μM Y27632 per well.
    **CRITICAL:** Co-culture medium must be warm to avoid dissolving the Matrigel BMM.
  h. Change medium with 500 μL pre-warmed co-culture media per well after 3 days. Addition of 10 μM Y27632 is not necessary.

### Co-culturing DRG and organoids for innervated organoids

**Timing: 30–40 min for DRG isolation, 30 min for preparing organoids and 30 min for plating co-culture**

This step describes how to isolate cervical and lumbar dorsal root ganglia (DRG) from newborn C57BL/6 mouse, to dissociate organoids, and how to seed intact DRG and organoids in Matrigel BMM for generating DRG innervated pancreatic organoids.

**CRITICAL:** Mice should ideally be aged between postnatal day 2 and 12. The DRG isolated from older mice have lower efficiency for neural outgrowth.

***Note:*** We advise to perform this step with two people. One person should prepare organoids while the other one is isolating DRG to minimize the time-dependent loss of cell viability.

**Figure 1:**
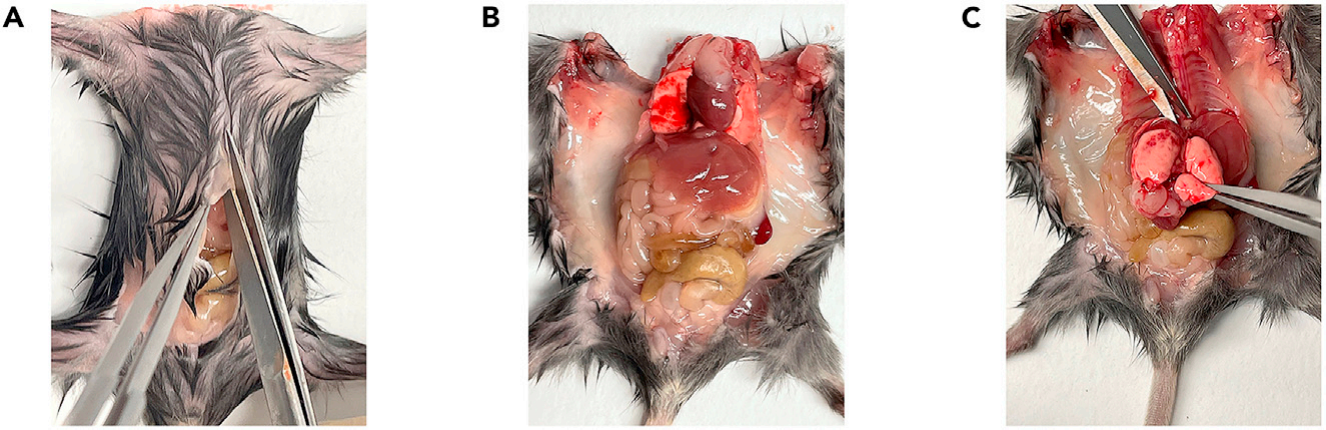
Dissection of the mouse (A) Laparotomy and thoractomy. (B) Exposed viscera of the mouse. (C) Removal of internal organs for exposure of the spine.

**Figure 2:**
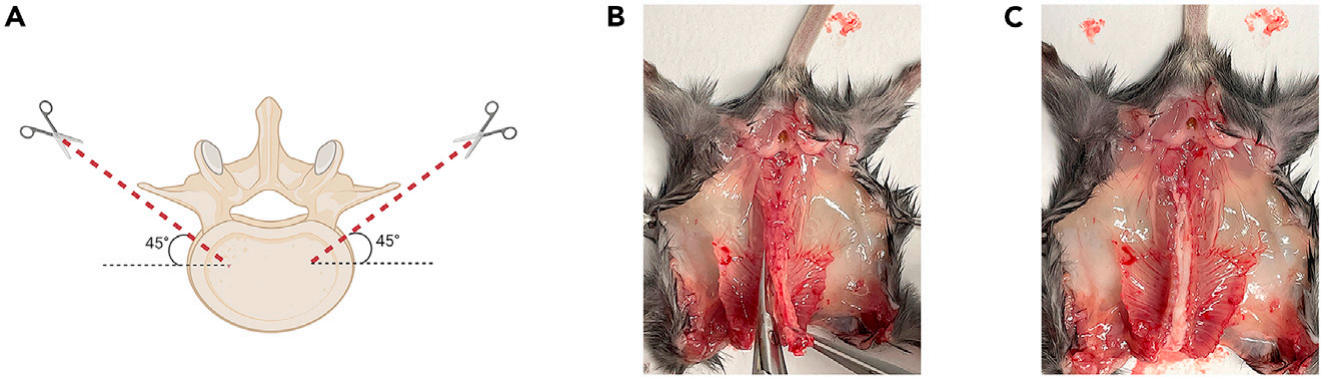
Technique of laminectomy during DRG isolation Schematic representation of anterior laminectomy angles (A). Performing anterior laminectomy (B and C). (∗Figure 2A was created with BioRender.com)

**Figure 3:**
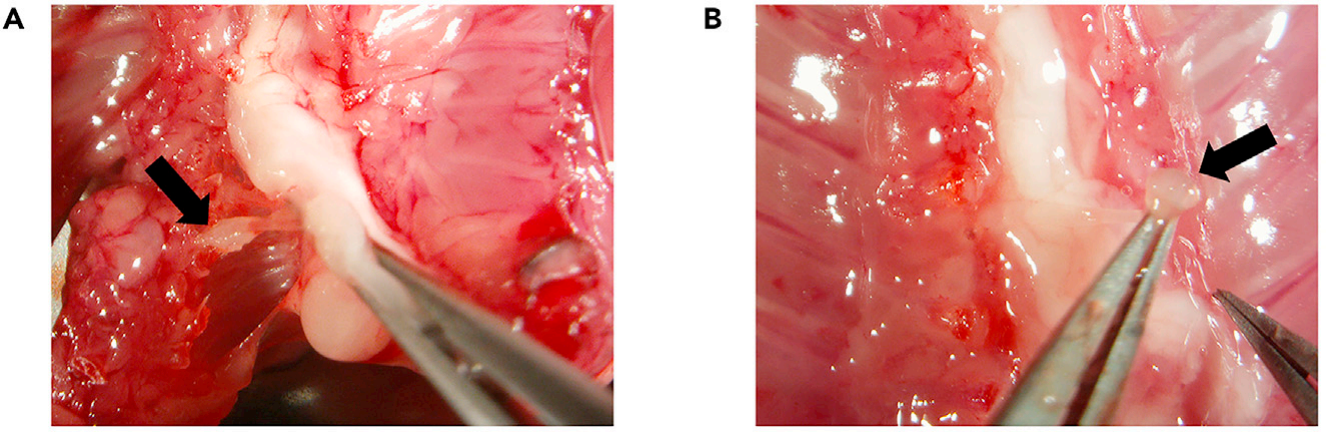
Removal of DRG from the spinal cord under the stereomicroscope DRG in the intervertebral foramina (Arrows (→) in A and B).

**Figure 4:**
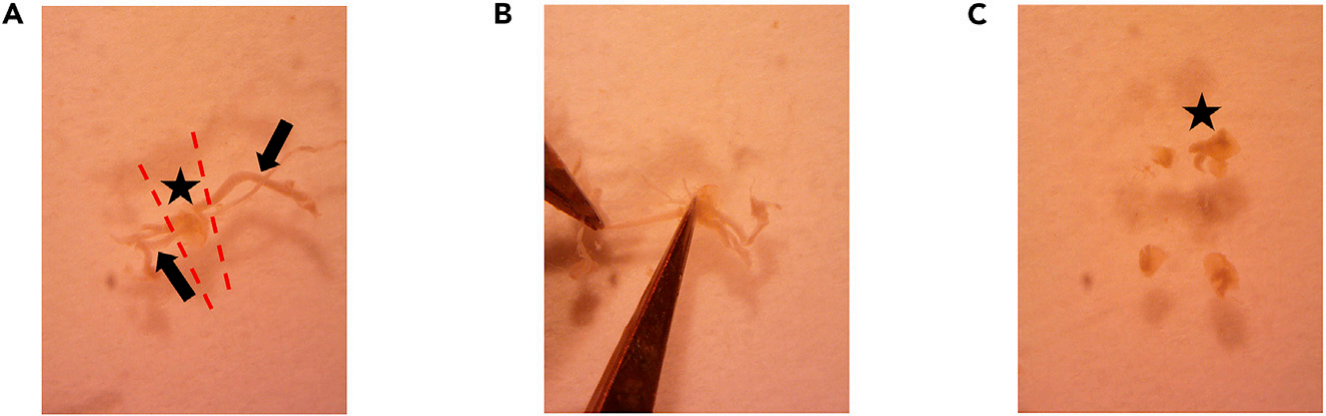
Removal of the projections from the DRG DRG body (∗) and projections (→) (A), cutting projections (B), intact DRG bodies (∗) (C).

9. Isolation of DRG from newborn C57BL/6 mouse.
  **CRITICAL:** Get the approval from national or local ethical committee for animal experiments and handle all procedure according to national ethics guidelines. Be careful to provide aseptic conditions as much as possible by working under a sterile dissection hood (clean bench cabinet with stereo microscope) and cleaning all the tools and surfaces with 70% ethanol. Neurons are very fragile. Be fast during this step and always keep the collected DRG on ice.
  a. Euthanize the mouse according to approved procedures.
  b. Clean the skin of the mouse with 70% ethanol and open the abdomen (Figures 1A and 1B).
  c. Take out all the internal organs (Figure 1C).
    **CRITICAL:** Be careful not to damage intestines because of the risk of bacterial contamination.
    ***Note:*** Continue the rest of this step with a stereomicroscope.
  d. Cut the lateral sides of spinal bone at a 45° angle by using micro-scissors and perform anterior laminectomy (Figure 2).
    ***Note:*** You can look at our previously published video protocol, which presents how to perform anterior laminectomy and DRG collection: Demir et al. (2014).
  e. Find DRG located in the intervertebral foramina (between vertebrae) and pick them up and directly transfer into petri dishes containing DRG collection medium on ice (Figure 3).
  f. Repeat Step 9/g. until you collect enough DRG for your experimental design from the cervical and the lumbar part of the spine.
    ***Note:*** A mouse has 16 cervical and 10–12 lumbar DRG. Try to collect as much as DRG you can and use one intact DRG for one 50 μL organoid Matrigel BMM droplet.
  g. Observe the collected DRG under a stereomicroscope and cut the peripheral and central projections out (Figure 4).
    **CRITICAL:** Cutting out the projections is very important to avoid contamination by fibroblasts which decrease the rate of neurite outgrowth.
  h. Transfer the DRG bodies into a new petri dishes, containing mouse organoid basic medium on ice.

10. Preparing Organoids for co-culture
  a. Repeat from Step 6/a to Step6/g.

***Note:*** Preparation of organoids is the same for both the co-culture methods. Preparing organoids from two wells in Step 6 and using them separately for NCCs and DRG co- cultures allows the comparison between innervated organoids co-cultured with NCCs (as neuro-progenitors) and co-cultured with DRG (as adult neurons) at the same time.

**Figure 5:**
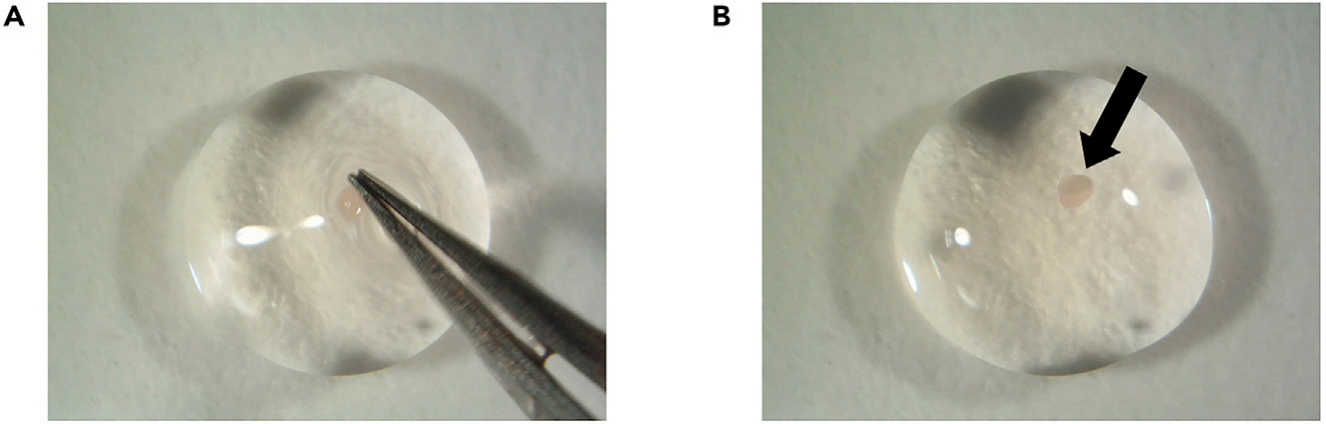
Embedding DRG into Matrigel BMM droplets Embedding DRG into Matrigel BMM droplets (A). Arrow (→) shows the DRG in Matrigel BMM droplet (B).

11. Preparing DRG and Organoid co-culture
  a. Make one 50 μL drop of Matrigel BMM organoid suspension in the one well of a pre- warmed 24-well plate.
  b. Immediately take one DRG body and embed it in the droplet with the help of sterile forceps (Figure 5).
  c. Repeat Step 11/a. and b. one by one for the other wells.
    **CRITICAL:** Perform these steps one by one and keep Matrigel organoid suspension on ice to avoid solidification of Matrigel.
  d. Incubate the plate at 37°C and 5% CO_2_ for 10 min.
  e. Add 500 μL pre-warmed co-culture media with 10 μM Y-27632 per well.
    **CRITICAL:** Co-culture media must be warm to avoid dissolving the Matrigel.
  f. Change media with 500 μL pre-warmed co-culture media per well after 3 days. Addition of 10 μM Y-27632 is not necessary.

## Expected outcomes

Applying this step-by-step protocol makes possible to generate innervation in murine wild type (WT) C57BL/6 derived normal pancreatic organoids and Kras+/LSLG12D Trp53fl/fl p48+/Cre (KPC) derived tumor pancreatic organoids by co-culture with neural crest cells (NCCs) or with dorsal root ganglia (DRG)-derived mature neurons. While the former represents the embryonic progenitors of the peripheral nervous system, the latter are mature neurons. Both models are very useful for investigating the underlying mechanisms of pancreatic neuropathy in pancreatic cancer. NCCs and organoid co-cultures can be an option for studies on pancreatic embryology. Furthermore, these co-culture methods may apply also to human pancreatic organoids.

**Figure 6:**
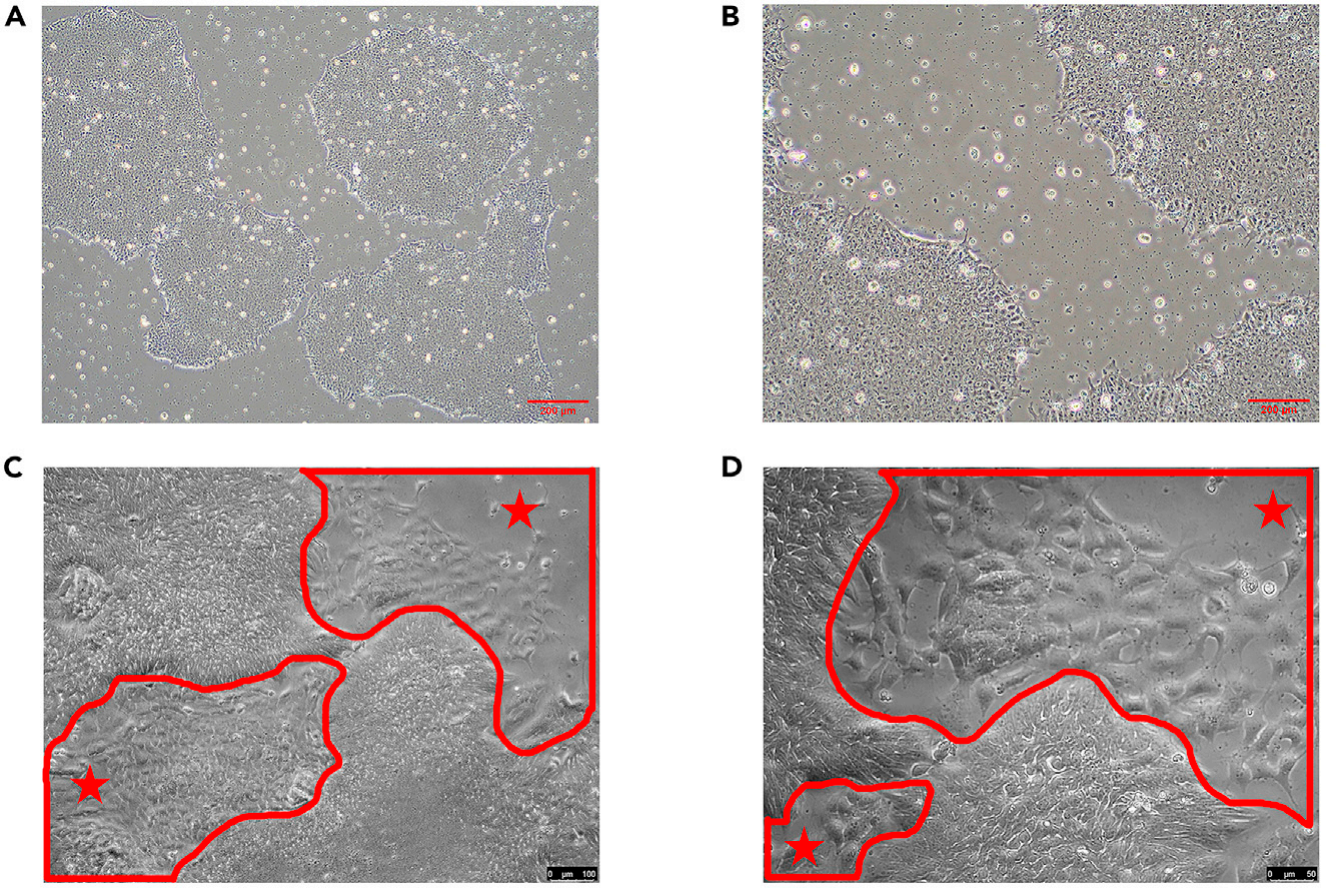
iPSC colonies Optimal compacted iPSCs colonies with smooth edges and without differentiation (Phase- contrast, A:4**×**, B:10**×**). Unwanted simultaneously differentiated areas around iPSCs colonies marked in red and star (∗) (Phase-contrast, C:10**×**, D:20**×**).

**Figure 7:**
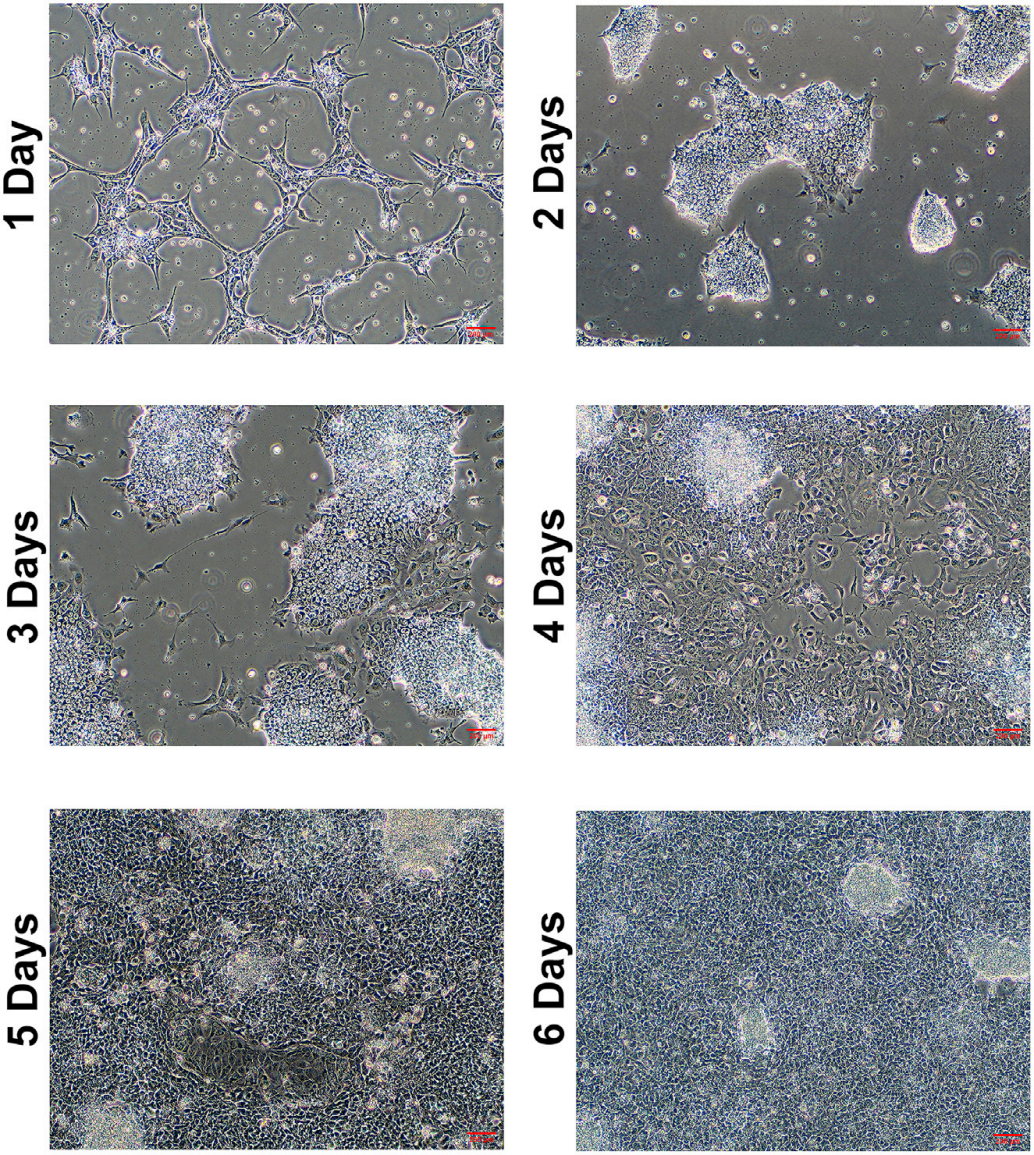
Daily morphological changes of NCCs differentiation from iPSCs growing in 6 well plates (Phase-contrast, 10**×**)

After 6 days of culturing, iPSCs colonies are ready for differentiation if that are compacted at the center and have smooth edges without any differentiation as shown in Figure 6. After that, iPSCs start differentiating to NCCs and their morphology changes day by day with an increasing confluency (Figure 7).

**Figure 8:**
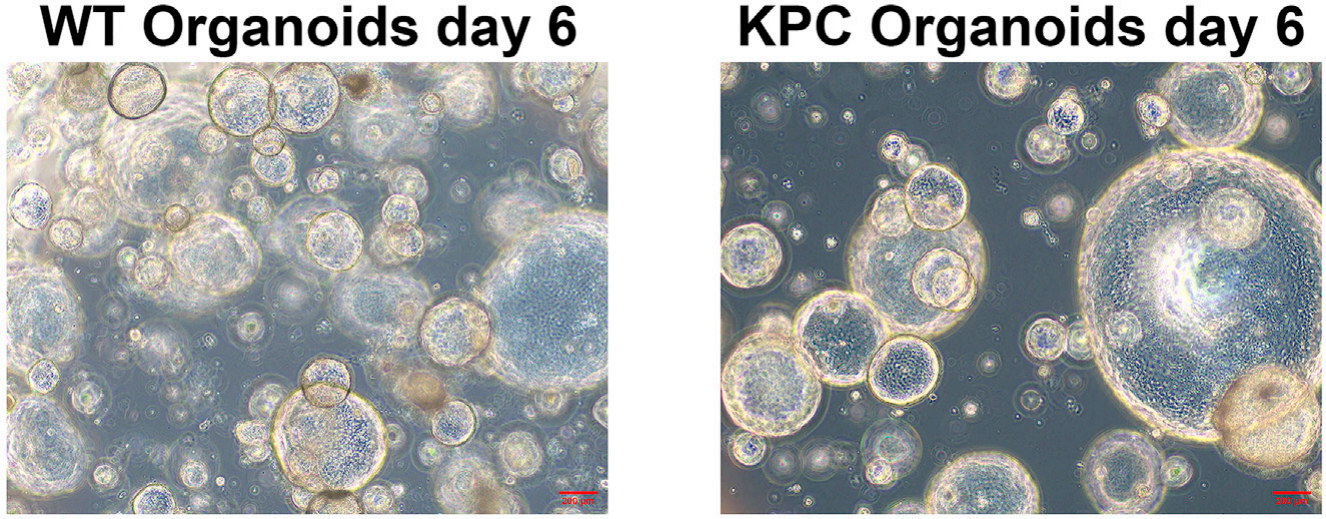
WT and KPC mouse pancreatic organoids at 80% confluency and ready for co-culture on the sixth day of mono-culture (Brightfield, 10**×**).

Organoids reach 80% confluency for co-culture 6 days after passaging them at a 1 to 6 ratio in 24- well plates. The size of KPC organoids is usually bigger than the WT organoids. (Figure 8).

**Figure 9:**
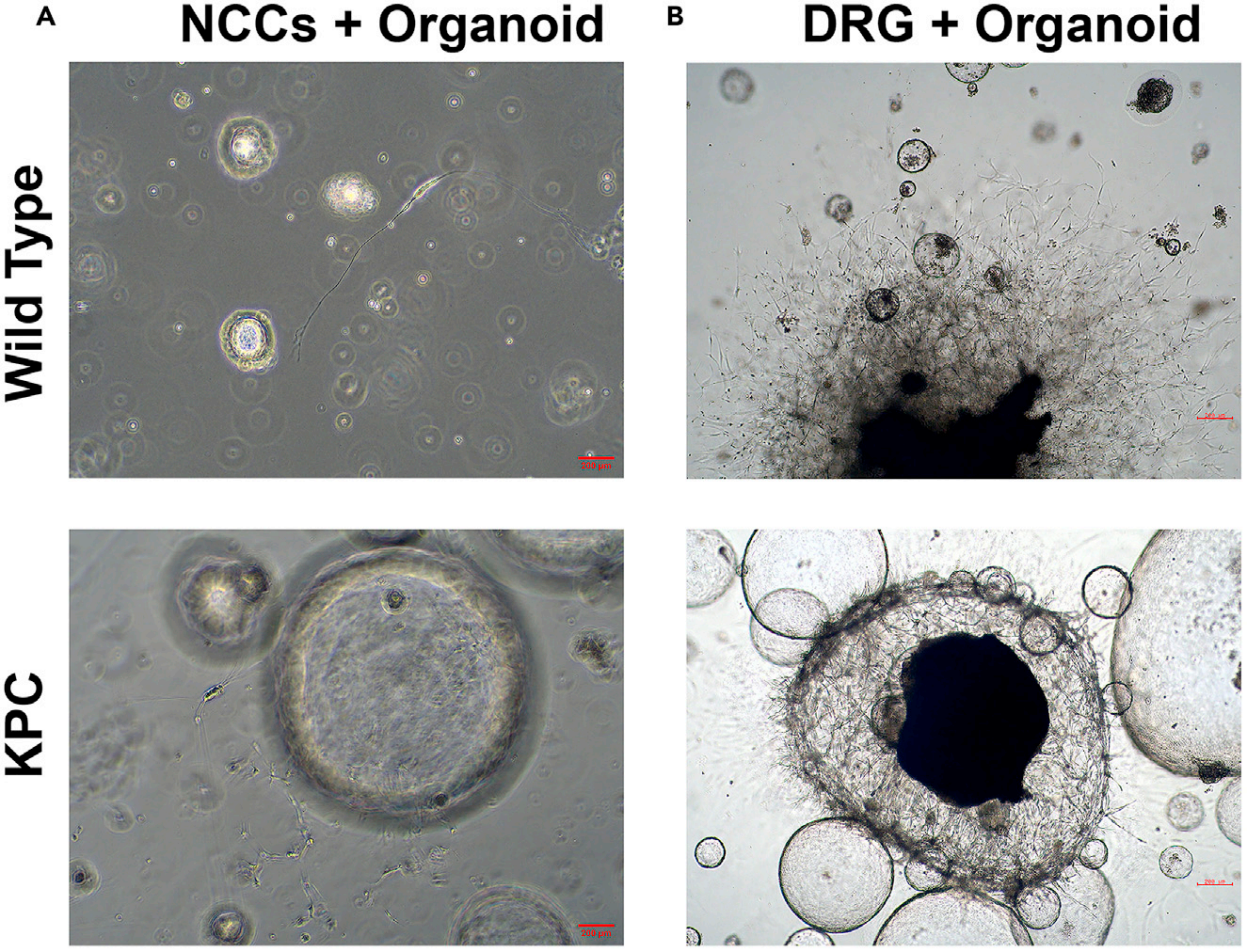
NCC, DRG and organoid co-cultures Differentiated neurons around the organoids in NCCs + organoid co-cultures (A. phase- contrast, 10**×**). Neural outgrowth from DRG to organoids in DRG + organoid co-cultures (B. brightfield, 10**×**).

**Figure 10:**
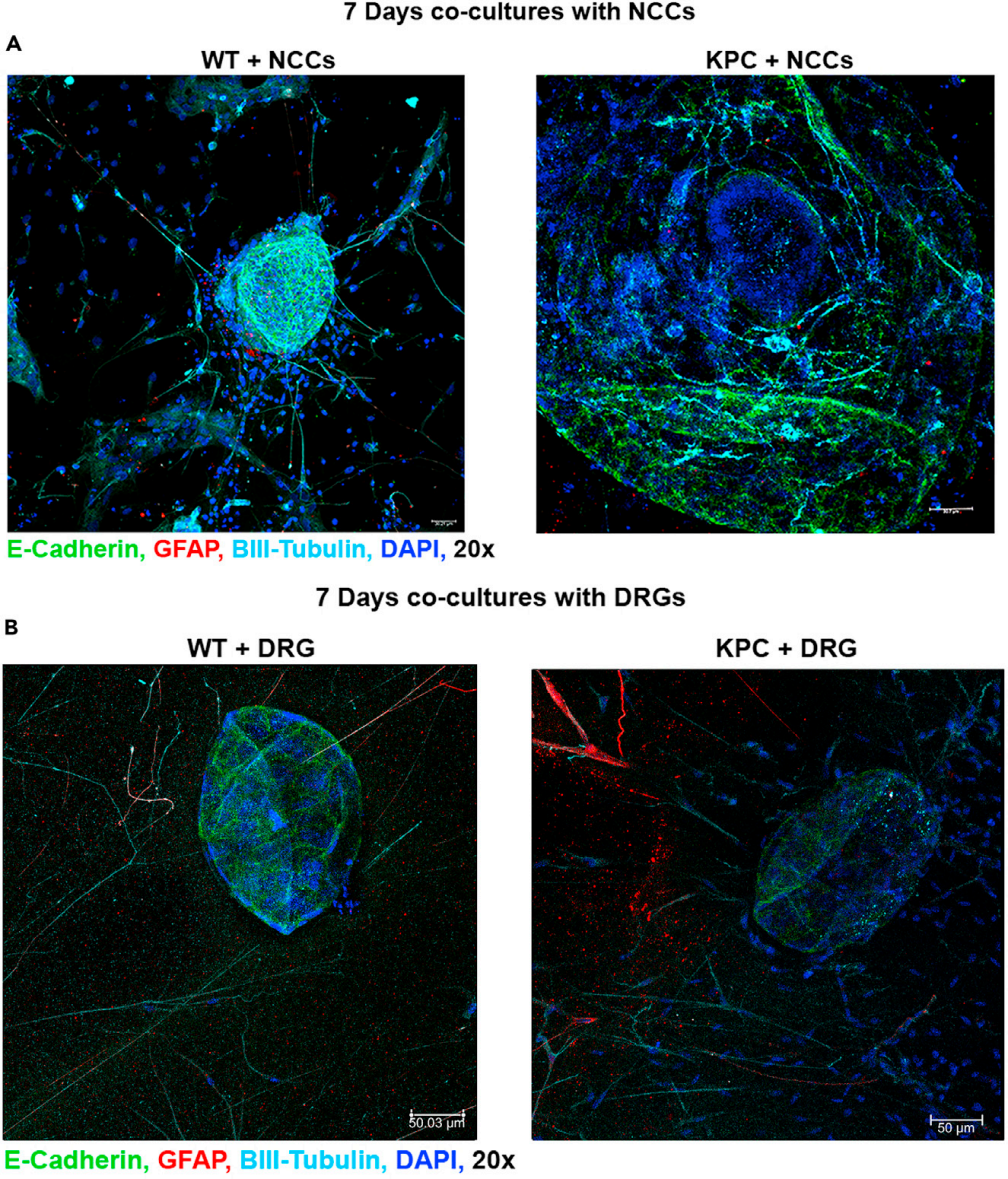
Innervated organoids Wholemount immunofluorescence staining of NCC + Organoid (A) and DRG + Organoid co-cultures (B). E-cadherin positive pancreatic organoids are marked in green, β3-tubulin positive neurons are marked in cyan and GFAP positive glia are marked in red. The nuclei are stained by DAPI marked in blue (Confocal microscopy, 20**×**).

NCCs starts to differentiate into neurons, and neuronal morphology can be observed in NCCs and organoid co-cultures after 3–4 days (Figure 9A). Neural projections start to grow out from the DRG towards organoids after 2–3 days (Figure 9B). After 7 days of co-culturing, B3-Tubulin positive neurons and some GFAP positive glial cells should be visible around the E-cadherin positive organoids with wholemount immunofluorescence stainings for both NCCs + Organoid co-cultures (Figure 10A) and DRG + Organoid co-cultures (Figure 10B).

## Limitations

NCCs differentiation potential of iPSCs can vary between cell lines because of the endogenous bone morphogenic protein (BMP) production. Innervation efficiency in NCCs - organoid co-cultures is directly related to the amount of harvested NCCs. Therefore, the starting iPSC line is very important for the success of the experiment and sometimes there is a need to try different iPSC lines.

## Troubleshooting

### Problem 1

iPSCs or NCCs do not attach to the plate.

### Potential solution

Coating the plates might have been unsuccessful. Use freshly coated plates. Check the dilution factor of Matrigel hESC-Qualified Matrix. Incubate the plates with coating medium more than one hour at 20°C–25°C. Do not open the incubator or disturb the cells for 24 h after seeding.

### Problem 2

Too many differentiated cells around iPSCs colonies.

### Potential solution

iPSCs colonies might have been dissociated too much. Avoid over-dissociation during passaging. Do not let the cells to wait outside the incubator for more than 15 min. Do not let the cells dry during daily media changes and add the medium very slowly on the side wall of dishes. Adding 10 μM Y27632 ROCK inhibitor in the medium can reduce the spontaneous differentiation.

### Problem 3

iPSCs are detaching from the plate during NCCs differentiation.

### Potential solution

Be sure your plates are coated properly (see problem 1). iPSCs has been stressed because of the over disassociation to make single cells and/or the time loss while passaging and seeding them for NCCs differentiation. Avoid too much disassociation and perform experiment as quick as possible. Add 10 μM Y27632 ROCK inhibitor into the STEMdiff^TM^ neural crest differentiation kit also on the second and third day of daily media changes.

### Problem 4

NCCs differentiation is not effective.

### Potential solution

Shorten the period between daily media changes such as 18 or 16 h after first day of differentiation. Try different iPSC amounts for differentiation. Prolong the differentiation culture up to ten days with daily media changes with the STEMdiff^TM^ neural crest differentiation kit. Try different iPSC lines.

### Problem 5

Too few or no neurons in the NCCs-organoid co-culture after one week.

### Potential solution

At least 50–60 normal pancreas organoids and 20–30 KPC pancreas organoids should be innervated in a successful experiment. KPC organoids are fewer in number, but they are bigger in size and occupy more space in droplet. NCCs differentiation from iPSCs has been unsuccessful (see problem 4). NCCs might be damaged during the preparation of co-culture. Avoid waiting too long between the steps. Use fresh co-culture medium with 10 μM Y27632 ROCK inhibitor on the first day and change medium on the third day. Try different NCCs and organoid amounts.

### Problem 6

Insufficient or no neurite outgrowth in DRG-organoid co-cultures.

### Potential solution

Almost all the organoids located as close as to the DRG should be innervated. Perform DRG isolation as fast as you can and do not wait between the steps. Be sure to keep the collected DRG on ice during the procedure. Connective tissue surrounding the nerve bundles of DRG projections reduce neurite outgrowth. Cut away as much projections as possible from the DRG bodies. Be gentle and avoid damaging the DRG while holding them with forceps. Use fresh co-culture medium with 10 μM Y27632 ROCK inhibitor on the first day and medium change on the third day.

## Resource availability

### Lead contact

Further information and requests for resources and reagents should be directed to and will be fulfilled by the lead contact, Ihsan Ekin Demir (ekin.demir@tum.de).

### Materials availability

This study did not generate new unique reagents.

## Data Availability

This study did not generate datasets or codes.
